# Mental health is everybody’s business: The doxa and illusio of local public policy implementation

**DOI:** 10.1177/13634593251399224

**Published:** 2025-12-04

**Authors:** Andrew Passey, Joseph Ibrahim

**Affiliations:** 1 School of Health, Leeds Beckett University, UK; 2 School of Humanities and Social Sciences, Leeds Beckett University, UK

**Keywords:** Bourdieu, doxa, illusio, policy implementation, mental health services

## Abstract

This paper provides a novel application of Bourdieu’s theory to the implementation of public policy in the mental health field, by deploying two of his lesser-used concepts (doxa and illusio). Framing a local case study as a Bourdieusian game, we demonstrate how these concepts have explanatory power within the field and how national policy discourses at once influenced and constrained by neoliberalism have real and long-lasting effects when it comes to the implementation of public policy. We found three key themes in the case study: doxa as a narrative device (‘mental health is everybody’s business’); the linking of this doxa with the game’s illusio in a shared view that the policy should address the problem of a high rate of ‘inappropriate referrals’ into specialist services; and the consequences of the dynamic between the game’s doxa and illusio for the organisational hierarchy in the mental health services field. We do not evaluate the success of the policy, but instead apply and explore the potential of Bourdieu’s concepts to provide explanatory power to the processes and interactions evident in the implementation programme. This paper highlights the potential of such a theoretically based analytical approach, which could be applied in other contexts and to other health fields and policy domains.

## Introduction

This paper applies Bourdieu’s lesser used concepts of *doxa* and *illusio* to understand implementation of policy in a local sector of the wider mental health field in England. Compared with the use of concepts such as field, habitus and capital, there is a relatively limited literature using *doxa* and *illusio*. Rather than implementation research, this tends to be situated in the educational field (e.g. Rowlands and Rawolle, 2013; Thomson, 2005). Organisationally, this field includes the National Health Service (NHS), local government and, as this paper outlines, an increasing role for schools and voluntary organisations. We argue that wider neoliberal drives for efficiency restructured and reoriented goals that underpinned a local doxic belief that the mental health of children and young people is ‘everybody’s business’. This paper focuses on a local case study of implementation of Future in Mind (Department of Health, 2015), hereafter FiM – a national (England-wide) policy that aimed to improve young people’s access to mental health services – to demonstrate how this belief had its roots in what Bourdieu (1996) calls the field of power, since it was drawn from national policy discourse by influential local actors, who used it as a narrative to shape local implementation of the policy in ways that expanded their reach into the education field (Bourdieu and Waquant, 1992). It was operationalised by the field’s logic of practice (Bourdieu, 1992) and manifested in players having a shared stake to reduce the referral rate into specialist mental health services.

This local doxa should not be seen as in any sense natural. Instead, its use by dominant local actors can be viewed as an active attempt to construct a new common sense in the dynamic and changing field conditions wrought by the insertion of a new policy (FiM). Following Bourdieu, we conceptualised implementation of FiM in Study-borough as a ‘game’. Bourdieu used the analogy of a ‘game’ to elucidate how actors compete in the field, in which they possess forms of capital that they use to maintain or gain power and influence (Bourdieu and Waquant, 1992). As this paper elucidates, in Study-borough, local actors had connected interest in the game, in the stakes they had in playing it and in the game’s consequences (Bourdieu, 1998). While the game’s specific manifestation was locally determined and therefore contingent, it was emblematic of historically-determined conditions in the field in which the local game was being played and of historical struggle between dominant and less powerful actors (Crossley, 2003). Nationally, this field was open to external critique. Reductions in local authority spending on youth and community services since 2010 (National Audit Office, 2018a), along with the growing number of children and young people being diagnosed with a common mental disorder and historic relative underfunding, had increased the pressure on specialist mental health services and contributed to negative public perceptions of those services.

The main argument of this paper is to demonstrate how Bourdieu’s concepts of doxa and illusio have explanatory power within the mental health field and thus have been reoriented to demonstrate how national policy discourses at once influenced and constrained by neoliberalism have real and long-lasting effects when it comes to the implementation of public policy. This is important as it highlights key power dynamics which are far reaching into a variety of fields, including those of health and education. This theoretically based analytical approach could be used in other policy arenas in addition to health care, for example, education or the criminal justice system. By employing the framework, researchers could uncover hidden assumptions, power structures and motivations, which shape such policy decisions, including but not limited to how actors set targets or produce data based on established practice that may not reflect real outcomes.

The paper is structured in the following way: firstly, we outline what we mean by a neoliberal doxa and an accompanying illusio, which emanates from the field of power and cascades down to all other fields, including the medical/ mental health field. Secondly, we discuss the local case study and methods used to make our case, after which we present our data and conclusions. We argue that Bourdieu’s concepts, in our view, have been under-utilised and yet offer explanatory power to understand how national policy as a narrative device in order to set agendas and achieve efficiency outcomes which have problematic consequences.

## Neoliberal doxa and illusio

Bourdieu’s concepts of habitus, capital and field are arguably the most utilised from his repertoire of thinking tools when it comes to explaining the reproduction of power, social inequality, and class distinction (Bourdieu, 1984, 1996). They are drawn on extensively and applied by Bourdieu and Bourdieusian scholars to explain how certain fields, amongst others and especially the education field, create an illusion of how success is disguised as natural talent. In reality, possessing the requisite dispositions (habitus) and embodiment of capital are the mechanisms for success, which in turn reproduce power relations throughout society (Bourdieu and Passeron, 1996; Friedman and Laurison, 2019; Reay, 2017; Savage, 2015; Waller et al., 2017). In this paper, we turn our attention to how a neoliberal doxa and accompanying illusio, prevalent throughout the mental health care field (a sub field of the wider medical field) has become the common sense and taken for granted practice for organising and running institutions.

A brief outline of what Bourdieu meant by both doxa and illusio will be instructive here. Doxa, for Bourdieu (1977), refers to the taken for granted, a set of unquestioned and undiscussed beliefs, which are manifested throughout our socialisation. Within institutions these beliefs are embedded more formally within everyday policies, actions and ways of being and thinking, to such an extent they are presented as the best, most efficient and sometimes the only way of practice. As Bourdieu outlined, doxa is ‘a particular point of view, the point of view of the dominant . . . [that] . . . presents and imposes itself as a universal point of view’ (Bourdieu, 1998: 57). If we take as an example the medical social world or what Bourdieu calls a field, the order and functioning of that world will be perceived by most as the normal and natural way of doing things, even though most accept changes to rules and practices over the years because there is an understanding to update certain practices – which of course, is ‘common sense’. Indeed, it follows, generally speaking, that on a day-to-day level most people will not question the functioning of that field, but rather just go along with it. This is not to say there is not criticism or a wish for change, but, by and large, the functioning of the field is accepted, perhaps, but not always, until a ‘real crisis’ occurs. In essence, most people follow procedure, obey authority, accept the delegation of duties and conform to both visible as well as invisible hierarchies of power. This, Bourdieu (1977) called, ‘ontological complicity’, because the order seems natural. The concept of illusio accompanies doxa, in the sense that it refers to how actors with their concomitant capital and habitus become invested in the field and its rules, they develop a stake in the field. Moreover, they believe it to be the way in which things should be and through their various actions reinforce the order of things.

Our contention is that neoliberalism, the dominant political and economic doctrine of successive UK governments, has influenced the structures and rules of most fields within society, down to what resources are valued and how actors within their respective fields act. Neoliberalism is the doctrine that suggests most if not all goods and services be marketised, and ‘market principles be allowed to permeate all aspects of life’ (Standing, 2011). This ideology suggests that markets are the most efficient means for producing the ‘best value’ and thus ‘best results’ for goods and services. On the ground within organisational practice, this often entails the implementation of a managerial structure that rationalises resources (including staff) to reduce costs and maximise outputs. Such changes can be brought through the altering of workers contracts to increase their workloads, outsourcing certain services or selling off certain services that are deemed expensive or profitable.

### Neoliberalism in the mental health field

The mental health system is a distinctive field, with its own logic of practice, in respect of having its own rules, resources and actors, each with their individual habitus and capital, but more importantly there is an institutional habitus, in this case a medical one. In short, despite it being heterogenous and comprising actors from different professions and organisations, all the actors understand, create, and agree the rules of this field and at the same time help to create and maintain it by their belief in it. Research has pointed to the mental health field becoming increasingly medicalised (Callaghan et al., 2017; Pilgrim, 2012). One consequence has been a shift from a notion of distress to one of illness, which is reinforced by economism discourses emphasising the financial costs of mental illness, the ‘burden’ it imposes on the economy and discourses that orient the mental health field towards individualistic and marketising responses to illness (Callaghan et al., 2017; McDaid and Park, 2022). Organisationally, this field exhibits a wider split in English health services between the commissioners and providers of services, as well as tariff payments for some medical interventions provided by health care organisations (Dopson, 2009). Both are emblematic of managerialist and performance measurement orientations in neoliberalism.

In this paper, we deploy two of Bourdieu’s less commonly used concepts in the field of implementation research (doxa and illusio) as explanatory tools to address three interconnected research questions: What was the orthodox and commonsense view (doxa) of the way to implement the policy locally? How did it influence and interact with the shared stake (illusio) that local actors had in playing the game to implement FiM? What were the consequences of this interaction for the pre-existing organisational hierarchy in the mental health services field? Besides providing rich empirical insights, our paper extends Bourdieusian analysis of policy implementation through testing the explanatory power of his lesser-used concepts.

## The study

### Policy context

Many nations, including the United Kingdom (UK), face the significant health and policy challenge of children and young people’s mental health (World Health Organization, 2021). In the UK, the COVID-19 pandemic cemented an upwards trend in prevalence of mental illness in children and young people. In 2023, about one-fifth of children aged between 8 and 16 had a probable mental disorder. In England, prevalence rose between 2017 and 2020, since when it has been broadly stable (NHS England, 2023). Evidence suggests that longstanding pressures on services to respond to this increasing demand are being intensified by gaps in the mental health workforce (British Medical Association, 2023; Royal College of Psychiatrists, 2024). As elsewhere in the NHS (Storey et al., 2019), one theme in English responses has been to shift some services into community settings, including a whole school approach to children and young people’s mental health and wellbeing (Department of Health, 2015; Department of Health and Department for Education, 2018; NHS England, 2021). Interventions include Mental Health Support Teams (MHSTs) in education settings involving new mental health practitioner roles in schools, and the commitment to train senior mental health leads in all state-funded schools in England by 2025 (NHS England, 2021). One foundation of this ongoing approach was FiM, a policy that received £1.4 billion of state funding between 2016 and 2021 (National Audit Office, 2018b). The policy was loosely designed, with broad national aims of improving young people’s access to emotional wellbeing and mental health services, reducing stigma, developing the mental health service workforce and increasing school-based services (Department of Health, 2015). Currently, there is only limited data about the impacts of FiM, with reviewers concluding that a lack of specific Government objectives and accountability mechanisms for the policy mean its national-level impacts remain unclear (Children and Young People’s Mental Health Coalition, 2023; National Audit Office, 2018b).

At this point, it would be helpful to situate the local game to implement FiM within the national policy context. The key policy texts were: *Future in Mind* (Department of Health, 2015), which was the work of the Children and Young People’s Mental Health and Wellbeing Taskforce established by Norman Lamb, a junior health Minister in the then Coalition government; the *Five Year Forward View for Mental Health* (Mental Health Taskforce, 2016) that included a recommendation to government to accept in full the FiM report; and the Government’s formal response in which it accepted that recommendation (HM Government, 2017). These texts can be read as ‘social events’, in which specific discourses act as ‘ways of representing aspects of the social world’ (Fairclough, 2003: 215), in this case the policy problem of children and young people’s mental health and wellbeing and options for policy responses.

There is a wide-ranging literature on policy implementation, a relevant strand of which for this paper focusses on the longstanding problem of an implementation gap in public policy and public service delivery (Osborne, 2010), that is a gap between the intent and articulation of policy and how it is implemented in service delivery. A lack of recognition among policymakers of the realities of ‘implementation in dispersed governance’ (comprising frontline and other staff and coalitions of different organisations) is one of a number of perceived factors that might contribute to the ‘failure of policy’, alongside over-optimism, limited collaboration in policymaking and changes emanating from the political cycle (Hudson et al., 2019). To bolster the chances of policy success, more open policy design has been posited to enable space for change, learning and innovation (Durose and Richardson, 2015). This would, in effect, seek to ‘design in’ discretion into how policy comes to be implemented and foster co-production. Others have argued for ‘implementation-minded’ policy that would be more mindful of what is required for ‘effective implementation’ and would acknowledge that ‘success depends on the support and activity of a range of actors, depending on where implementation takes place’ (Baan et al., 2023). This literature makes clear that policy implementation is complex and not linear or top-down (Hudson et al., 2019). These points, that policy implementation requires a range of (potentially local) actors and that implementation is a complex process, provide important context for the study we outline in this paper. We do not however concentrate on the fidelity of local implementation to national policy intent or any notion of policy success (or failure). Instead, our focus is on how specific, local actors oriented policy implementation towards a locally-constructed view of who should be involved in delivering the policy and a common-sense view of the local problem and required response (the joint interest of those involved).

FiM itself was a loosely designed policy, although there were explicit attempts at the national level to frame local implementation within an existing national – local hierarchy. The FiM report claimed it had ‘described a vision for our country in which child mental health and wellbeing is everybody’s business’ (Department of Health, 2015: 73), while the subsequent report that took forward FiM argued ‘[d]elivery . . . is everybody’s business - for the NHS, for health and social care professionals, for providers, employers, across government and communities’ (Mental Health Taskforce, 2016: 20). Further, these national policy texts positioned local NHS commissioners of mental health services as leaders in the drawing up of local plans to implement FiM, in the process helping to cement their status among the ‘dominant’ local actors. The emergence of mental health being everybody’s business as a local doxic belief can thereby be seen to align with the national policy intent.

Additionally, these national policy texts foregrounded social practices in discourses associated with neoliberalism. Far from being a factual statement, the directive that mental health is everybody’s business can be read as emblematic of neoliberal perspectives. The medicalisation of emotional distress was evident in the unqualified use of ‘mental health’ as a fixed category, although the meaning seems more specifically to relate to ‘poor mental health’. The claim of it being ‘everybody’s business’ responsibilised different organisations in tackling it, and potentially abstracted individual children, young people and families from their social contexts to responsibilise them for ameliorating their own emotional distress. ‘Business’ pointed to an economism discourse. In combination, these neoliberal discourses served more dominant interests by naturalising and creating as taken-for-granted conditions of the game that were actually arbitrary (Bourdieu, 1977; Fairclough, 2003). The imparting of national discourse as local doxa had a structuring power that made it influential in how the game was set up and run, and in how the policy was locally implemented. This doxic view was, in effect, the dominant view in the game.

### Research context

The case study site was Study-borough,^1^ a mixed urban and rural area in northern England. It has a population in the range of a quarter to half a million people, and the proportion of its children who live in poverty is just above the national average. The 2019 Index of Multiple Deprivation ranked it among the 20% most deprived areas in England. In Study-borough, local implementation of FiM involved a new partnership of health commissioners and service providers, local authority services, counselling services and voluntary and community organisations (VCOs) operating in youth and community services, mental health services, support for recovery from addiction, and advocacy. This combination of organisations had potential to significantly extend the locally-constructed mental health services field well beyond the NHS, and in doing so reflected more ‘[e]mergent health and well-being perspectives’ seen in other parts of the UK health system (Storey et al., 2019: 190). Local actors reported a high rate of referrals into specialist services, which fuelled long waiting lists. Engagement by local actors in the new partnership was in part driven by a need to ease these pressures on Children and Adolescent Mental Health Services (CAMHS).

### Data collection and analysis

The original case study on which this paper draws (Passey, 2020) deployed a purposive sample design (see Table 1), a non-random approach that involved identifying and recruiting individuals whose experience and knowledge made them most relevant for the study (Creswell and Clark, 2017). Access to key participants was facilitated in an intensive period of fieldwork in the case study site. The lead author engaged potential participants at local meetings about the programme (e.g. the formal programme launch, partnership meetings, organisational team meetings) to explain the nature and scope of the study, and tested potential interest in participating in the study. The lead author’s presence at these events was initially facilitated by academic and practitioner colleagues, which lent the sampling strategy an element of convenience (Metz et al., 2022). Having ascertained potential interest, the lead author followed up by email. In this paper, we report on analysis of 26 semi-structured interviews undertaken with local actors involved in implementing the policy (see Table 1), 22 of which were with actors who identified as female. The sample was rich for most groups, in respect of the proportion of potential interviewees in each sector who were in the final sample. The lead author’s host university granted ethical approval for the study.

**Table 1.** Interviews conducted in the study.

Participants	Interviews	Population size	Pseudonym
Commissioners: (NHS England regional; NHS - CCG policy area)	4	6	Comm
CAMHS staff (team leaders/service managers, community-based practitioners)	8	13	CAM
Local authority staff/other providers	5	10	LA/Other
VCS coordinators, managers, trainers, community-based staff)	9	18	VCS
Total number of interviews	26		

Fieldwork lasted from September 2017 to January 2019. All interviewees gave their written informed consent. Interviews were undertaken face-to-face in participant workplaces, except for one telephone interview and lasted just under 1 hour on average. A semi-structured interview topic guide ensured consistency of coverage, while allowing space for emerging issues. The guide asked participants about their work and experiences in FiM, with whom they worked, the nature of those working relationships and for their perspectives on collaborative working and co-production. Quotes are attributed to professional/sector groups, rather than individuals, to preserve anonymity while distinguishing different sectoral views.

The wider study on which this paper is based (Passey, 2020) involved a thematic analysis (Braun and Clarke, 2006) to analyse interview data, supplemented with data from 10 observations of meetings during the fieldwork process. Interviews were semi-structured with a standard topic guide covering participants’ views on their work in the implementation programme (which could be at service or programme level). It also sought their views about the programme to date and its prospects, and explored participant perspectives about the programme’s commitment to co-production and what that meant in practice. The lead author had used NVivo software to transcribe these interviews, organise the data, inductively code (the coding had not been informed by any pre-existing coding framework) and develop themes. These coded data were then exposed to theory, including theories of co-production in public services (a key motivation for the original study) and Bourdieusian theory, specifically his concepts of doxa and illusio (the focus of this paper). In this iterative process of ‘theoretical re-description’ (Fletcher, 2017) we generated a theoretically-informed interpretation of the story of the local game that provided further explanatory power to the case study.

Table 2 summarises this process in relation to the three themes we generated through our use of Bourdeiusian concepts. To explore the game’s doxa, coded data were examined for excerpts referencing underpinning rationales for the multi-organisational approach in Study-borough. One feature that emerged was excerpts including the statement that ‘mental health is everybody’s business’, the operationalising of which was coded as an aim of FiM in Study-borough. In particular, CAMHS staff and NHS commissioners most readily articulated this statement in making a case for involving a new collaboration in implementing the policy. These excerpts, plus specific quotes where local actors described how the programme was managed, its impacts and its limitations, were re-coded as representations of the game’s doxa. To explore the game’s illusio, coded data were examined for excerpts pertaining to the key goal or ‘win’ for FiM in Study-borough. Specifically, references to the high rate of referrals, especially inappropriate referrals, emerged as both the key local challenge and, if the rate could be reduced through FiM, as a potential local prize and as an impact of the programme locally. These data excerpts were re-coded as referencing the game’s illusio and were gathered from interviews by actors from across the range of organisations involved in implementing the policy. The data also included perspectives on changes in the field, which we coded into a third theme ‘consequences for the organisational hierarchy in local field’. While not specifically about doxa and illusio, this theme provides some sense of the local game’s dynamism.

**Table 2.** Generating theory-based themes summary.

Inductive codes that provided recoded data		Bourdieusian theory-based themes
Aims of FiM	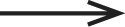	Doxa as a narrative device
Impacts of FiM: cross-organisational		
Programme management: partnership coordination		
Impacts of FiM: service/organisational	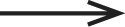	Linking of doxa with the game’s illusio
Impacts of FiM: cross-organisational		
Aims of FiM		
Limitations to FiM		
Local context		
Impacts of FiM: cross-organisational	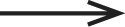	Consequences for the organisational hierarchy in the local field
Sustainability: new ways of working		
CAMHS organisational inertia		
Limitations to FiM		
Local context		

Because we were in effect recoding data from within the original inductive codes, there was no straightforward translation of all the data in an inductive code to the more conceptual, theory-based themes. Instead, different data from one relevant inductive code could be recoded into different themes. For example, excerpts originally coded to indicate ‘Limitations to FiM’ contributed data items to two of the theory-based themes. Eight of the original study’s 23 inductive codes contributed data to the Bourdieusian theory-based themes that we discuss in more detail below.

## Findings

We generated three theory-based themes in our analysis. First, was a narrative device that sought to broaden the range of actors responsible for implementing the programme by propagating a view that ‘mental health is everybody’s business’. This doxa was specifically used by dominant interests in the game (commissioners who funded the programme and specialist mental professionals who exerted strong influence over practice in the field) and had been cemented in the game through activities by these two groups of actors. Second, was the linking of this doxa with a shared view of the problem that needed to be solved in Study-borough, which was to reduce the rate of what were termed ‘inappropriate’ referrals of children and young people into specialist mental health services. This determined what was at stake for the extended range of actors playing the local game to implement FiM, becoming the game’s illusio. Third, were consequences of the dynamic between the game’s doxa and illusio, manifested in changed activities and service outputs, and more fundamentally by the reproduction of the pre-existing organisational hierarchy in the local field. In short, the articulation of a common-sense view of who should be involved in FiM, and what they shared as a stake by being involved, served the interests of dominant interests in the local field. This was despite the introduction of FiM disturbing specific features of the field, such as through the insertion of new organisational actors.

### Doxa as narrative

Local actors based in commissioning organisations and specialist mental health services propagated a view that ‘mental health is everybody’s business’. In so doing, they were echoing a theme in national discourse about the shape of policy responses to the problems of children’s mental health (Department of Health, 2015; Mental Health Taskforce, 2016). This statement is clearly universalising in a strictly syntactic sense. However, when deployed as a universal notion in the game it gained a broad legitimacy among all actors. Its meaning was much deeper than the purely syntactic, and in consequence it had potential to shape, adjust and structure the perceptions of players in the local game (Bourdieu, 1984: 471). Rather than using this view as a metaphor, these actors used it as an expression of the scope of interests they wanted to incorporate into local implementation of FiM, such as local voluntary organisations and, most critically, schools. As local actors explained, one of the programme’s aims was ‘about getting the message out there that mental health is everybody’s business’ [CAM] involving active efforts to ‘get that message across . . . that it’s everyone’s business’ [Comm]. One participant expressed this as a concern . . . ‘what we’re trying to say is “we’ve all got mental health. Everybody’s got mental health”. And but for the grace of God, you don’t know what’s going to happen’ [CAM]. Children’s mental health needed, in effect, to be normalised as an issue that warranted attention from a wide range of organisations across Study-borough. This was a key basis for activity in the local programme, as a commissioner explained . . . ‘actually our approach backed up the way we’ve taken it, because it’s all young people at all times actually that’s everybody’s business’ [Comm]. Fundamental to this effort was making a compelling case to local schools, which were viewed in the programme as ‘change agents . . . because children spend an awful lot of time in school’ [CAM].

This work was complicated by neoliberal reforms, which meant that schools operated in a marketised environment. Their reputations were founded on performance in examination results league tables and external inspections, itself reflective of an audit culture in public services (Ferlie and Geraghty, 2005). Work to engage schools in FiM needed to negotiate this context. Much of the initial activity by commissioners and managers required work to get school leaders and senior teachers to recognise a link between the emotional wellbeing and academic performance of children and young people, and as a consequence of that recognition, to engage fully in the programme. This was a unifying argument that cut across all schools, given they were subject to Ofsted inspection. The appeal was thereby consciously designed to align with, and support, a primary motivator of schools. Participants explained how they went about relaying a narrative to schools about what FiM had to offer:

Saying the benefit for that is, one is to the young person, two is to you directly because you’,ll have better educational outcomes, you’ll have happier young people, you’ll have better attendance. [Comm]

Another participant from specialist mental health services described it in detail:

I think what we’ve been trying to do is make that kind of distinction, that the two aren’t mutually exclusive. That in order to actually have children who achieve . . . academic excellence, then they have to have those things in place that mean they’re happy and well, both physically and mentally. So actually, a school that is, has got a kind of good emotional wellbeing, pastoral, kind of, ethics, will also kind of see that come out in achievement as well. [CAM]

In summary, this was how schools were convinced of the wider notion that children and young people’s mental health was everybody’s business, including their own. While this work had taken time and much of the initial energy of the programme, there was reportedly complete sign-up of in-scope schools by the time fieldwork was being undertaken in the case study. To an extent at least, that indicated the legitimacy of the notion that mental health was everybody’s business. It had acquired the status of a *doxic* belief in FiM in Study-borough.

Realising *doxa* through *illusio*: cementing a view about inappropriate referrals The legitimacy conferred in the *doxic* belief that mental health is everybody’s business endowed it with some power over the investments that local actors had in playing the game in Study-borough. Central to this investment what was described as the ‘significant number of referrals’ [CAM] of young people into specialist services in Study-borough. The rate of referrals was deemed too high to be sustainable, given the strain it put on existing services and, critically for local services, on children enduring long waits for care. As another actor claimed ‘CAMHS being in crisis with our waiting lists . . . is very knowledgeable [well known] for everyone’ [CAM]. The local service did not enjoy a positive reputation, but instead ‘perceptions are really negative of the CAMHS service. It’s got long waits’ [Comm].

Actions intended to bear down on these waiting lists were central to the approach in Study-borough, although this service problem was further nuanced in the specific stake actors had in playing the game of FiM implementation in Study-borough. Core to this inflexion was a commonly-held view that at least some of these referrals were ‘inappropriate’, since they did not reach the threshold level for intervention by specialist mental health services. A concern over the volume of referrals was refined into a quality perspective which, in turn, emphasised a collective stake for all actors rather than for a single organisation. As one actor reflected of the situation before FiM, ‘we used to have a high inappropriate referral rate into CAMHS’ [Comm]. Local children and young people (and their parents or carers) were reportedly frustrated when they were not able to access specialist services without a sustained wait and FiM was identified as one way to improve their situation:

I think it [FiM] will really help to get appropriate referrals and hopefully stem the flow of families and children needing the more acute end of the services, that’s the aim. [LA/Other]

My understanding was that we were going to invert the triangle, and we were going to have more community-based interventions therefore reducing the need for referrals to CAMHS services at that higher level. [VCS]

Reducing the rate of inappropriate referrals was a core local priority. All players had a stake in making it happen. The adoption by all players of this shared stake was a vital manifestation of the local game’s doxa. It had a dynamic relationship with implantation by ‘dominant’ interests of a particular common-sense view and became a collective investment precisely because of the legitimacy of a *doxic* belief that mental health is everybody’s business.

The specific approach in Study-borough reveals the purchase of the game’s interlinked local doxa and illusio, since having more staff attuned to children’s mental health ran the risk of increasing activity as more children were deemed in need of support from local services. This seems counter-intuitive, given a shared local view that the level of referrals was already too high at the time FiM was introduced. The dynamism of the local game and of the explanatory power of Bourdieu’s concepts is clearly evident in the willingness of actors from across organisations in Study-borough to adopt the local approach and to actively engage in the programme.

The plan was that by making mental health everybody’s business and working to minimise rates of inappropriate referrals, services would eventually reach a state of balance. The programme involved more services than had been the case immediately before it started, many of them rooted in community-settings that were tasked with ‘ensuring that unmet need is met, and that, you know, people who may not access services do so as well’ [Comm]. In addition, increased awareness had the potential to stimulate the identification of greater need in the community, which in turn could lead to more referrals into statutory mental health services, not fewer. The proposal was that new services, coupled with greater awareness in non-specialist staff, would facilitate early intervention and prevention. Ideally, this would mean that ‘they [children and young people] don’t actually become so unwell that therefore they need secondary mental health care’ [CAM], which was more intensive, expensive and under severe demand pressure. The short-term dynamic was summarised by one CAMHS worker:

We were getting less referrals from schools, or we were getting more appropriate referrals. So it might be that our referrals went up, but actually it was more appropriate referrals, rather than. . . Because before schools might say ‘oh we’ll just have to refer to CAMHS, because what else are we going to do’. [CAM]

Reportedly, manifestations of the local game’s doxa and illusio were began to move referral rates in the intended direction, with the overall number of referrals and ‘inappropriate’ referrals into CAMHS starting to fall:

We have a done a lot of work, and FiM has gone a long way to doing this, around appropriate referrals. So we get a lot less appropriate, inappropriate referrals than we used to do. [CAM]

. . . the number of referrals into CAMHS has, has dropped. The number of inappropriate referrals has dropped because staff are being [given] the skills to be able to identify when a young person does need to be in CAMHS and identify when a young person just needs support within school. [Comm]

Reproducing the field’s organisational hierarchy

The doxic belief that mental health is everybody’s business shaped what players thought was possible within the rules of the local game and underpinned shared investments that players had in playing it. The ability of this doxa to shape the game stemmed from it being viewed by players as inclusive, universal, and ultimately, common sense. This belief manifested in more optimistic views on the sustainability of the changes from the programme. For example, commissioners felt that ‘it’s really, it’s important that the cultural aspect of what we do, and the ways of working, and that being embedded everywhere, is our safety net’ [Comm]. They pointed to changes in school behaviour ‘I think where we’ve seeing some examples of, where kind of work might be finishing in certain schools but schools are kind of maintaining a lot of it themselves’ [Comm].

Instead however of being the common-sense view of relations in the local game that it appeared, the local doxa had the effect of helping ‘dominant’ interests make and constitute the social reality of players in the local game (Loyal and Quilley, 2017: 432). This act of ‘misrecognition’ (Bourdieu, 1977) had its origins in the legitimacy that the doxa had for those who were playing, within the confines of the game’s rules. In practical terms, new organisations were stepping into the mental health field to serve interests of dominant actors:

I think that has really reduced now [referral rate] because . . . schools and universal services are recognising there’s lots of things they can be doing kind of in-house, which would be helpful for that child’s mental health and emotional wellbeing in more of a preventative way or more of an early intervention way [CAM].

Actors’ shared view of the legitimacy of the local doxa and the shared stake to bear down on inappropriate referrals served clinical, institutional interests. Specifically, the game allowed these interests to determine what made a referral inappropriate. In so doing, they were exerting another form of symbolic power in relation to less powerful actors, rooted in the legitimation of a particular form of cultural capital (Bourdieu, 1986). That symbolic power was evident in the definition of what was inappropriate, and the ways that specific case-by-case judgements were made in relation to the threshold levels for referring into statutory mental health services. FiM instigated new multi-organisational meetings to review individual cases. These forums were consistently viewed by local actors as a strong beneficial change wrought by FiM. Descriptions of the process did however suggest the leading role played by CAMHS practitioners, whether or not the case was ultimately referred to specialist services:

. . .we could have one-to-one consultations with the [CAMHS community-based workers]. So that enables us to do a number of things really. One, it made sure that, that referral was assessed appropriately as to whether it needed a full referral into CAMHS. So, by having that consultation, that decision could be made [LA/Other].

The thresholds to which CAMHS staff were working were not spelt out and remained opaque. There was no specific formula or scorecard. Instead, deliberation was described in one FiM meeting by a CAMHS practitioner as ‘a conversation, it’s the working it out, and if you’re not sure, it’s about asking the questions . . . [you] . . . can’t take away professional judgement’. In this way, the process remained unclear and professionally mysterious when viewed from outside. In this example, particular professional knowledge remained closed-off to other staff working in FiM. Actors based in specialist services maintained their monopoly power to define what made a referral inappropriate. In so doing, they were able to defend their existing advantageous position in the hierarchy, and by extension to reinforce the relatively weaker social position of less powerful actors.

Other actors revealed some awareness of this, claiming that CAMHS staff tended to use ‘medicalised language . . . it’s a power tool, isn’t it?’ [VCS]. We did not observe these actors directly challenging staff from specialist services on this issue in multi-organisation settings, but they appeared aware of the consequences of power imbalances in the programme:

. . .it’s really hard when you’re working with what is a statutory or a mental health service like that, for them to be able to adapt and make changes to what they’re already doing. I do think that element’s not been as successful [VCS].

They pointed to the scope of specialist services in the area as a limit to change that FiM might bring:

[We] . . . have all elements of the CAMHS service. So . . . whatever they do, whatever [CAMHS provider] does determines what, how the programme is . . . I suppose the worry is, is that the power of a big organisation like that can therefore influence not just, it can influence everything, commissioners and all of the partnership [VCS].

The overall effect was that specialist services exerted significant influence over the volume of traffic into specialist mental health services. This enabled them to focus more attention on cases of acute need, having regulated the flow of cases into specialist care services. At the same time, they were able to steer back to other, community-based services in FiM, cases deemed not to have met the CAMHS threshold and which were accordingly classified as inappropriate. This all had benefit to ‘dominant’ interests in potentially leaving undisturbed pre-existing hierarchical relations that underlay the game. Instead of them being upturned, these relations would continue to be reproduced in self-reinforcing ways (Deer, 2008: 121). Further, ‘dominant’ interests involved and in effect deployed other actors to reshape the game and the wider service system, but in ways that they themselves had determined.

## Discussion and conclusion

This paper provides a novel application of Bourdieu’s thinking tools to the implementation of public policy in the mental health field. It has outlined how a common-sense view (doxa), imported from national policy discourse, was actively used by certain local actors to help shape the implementation programme, in so doing extending the range of organisations involved in addressing the ‘problem’ of children’s mental health and wellbeing. This local doxa was linked with the shared interest actors had in implementing the programme, which formed the game’s illusio. Our aim was not to evaluate the success of the policy, but instead to apply and explore the potential of two of Bourdieu’s lesser used concepts to provide explanatory power to the processes and interactions evident in the implementation programme.

The game in Study-borough was itself being played within a collaborative logic of practice, exemplified by the new implementation partnership. Despite this having potential to problematise Bourdieu’s view that relations between actors in social space are predominantly based in competition, the use of two of his less commonly deployed thinking tools (doxa and illusio) provided a strong degree of analytical purchase. Broadly, they enabled examination of whether the local logic of practice challenged or reproduced the dominant neoliberal doxa in the local game. More specifically, they helped to explicate how the game was constructed in ways that effectively forged a degree of unity among those playing in it.

First, in conceptualising as a local doxic belief the notion expressed by local actors that mental health was the business of all players and not just specialist mental services, the ‘taken for granted’ was manifested. This is important in that the belief that became doxa is not rhetorical or metaphorical, but became concretised through practice, which at once highlights the reality of the empirical situation and the usefulness of Bourdieu’s underutilised concept.

Second, viewing a commitment to reduce the number and increase the quality of referrals as the illusio of players in the game revealed how they might have a shared interest in pursuing particular practices. These efforts at unity underpinned players’ stake in the game, which they tended to see through a professional, service-based lens. Work to underpin ‘relating as collaborating’ (Anteby et al., 2016: 227) was however somewhat limited as the boundaries between organisations both became more blurred, and the field’s organisational hierarchy was reproduced. This tension reveals the dynamic between the game’s doxa and illusio.

In sum, Bourdieu’s thinking tools provided a means for an exploratory analysis of a dynamic case of policy implementation. Doxa in this regard offers explanatory power since it captures practice which came into conflict with the professional belief in the game (illusio). In this sense doxa can be clearly read as a soft determinism, whereby the often unquestioning and subtle ways organisations and institutions work when implanting their policies negate individual professional thinking and understanding. That is, the mental health practitioners as players had to work within the already carved pathways of organisations and institutions, underpinned by policy implementation, thus treating everyday practice as ‘common sense’ and with the understanding that this is the way things work best, often with a pragmatic focus. This is not to claim an instrumental approach, rather, it is more a case of a sometimes pre-reflexive and often uncritical one; and even when criticism of practices is raised it sometimes has little or no impact until a crisis occurs resulting from forms of practice bringing the doxa to the level of discourse, which then may create new forms of thinking and practice to rectify any perceived weakness with organisations and institutions (Bourdieu, 1977). To this end, our paper highlights the potential of such a theoretically based analytical approach, which could be applied in other contexts, and to other health fields and policy domains.

## Notes

1. This is a pseudonym. The case is a local authority area in the north of England.

## References

[bibr1-13634593251399224] Anteby M Chan CK DiBenigno J (2016) Three lenses on occupations and professions in organizations: Becoming, doing, and relating. Academy of Management Annals 10(1): 183–244.

[bibr2-13634593251399224] Baan AM Lewis J Wills E , et al (2023) Implementation-minded policy making. Available at: https://www.wcpp.org.uk/wp-content/uploads/2023/11/Implementation-Minded-Policy-Making.pdf (accessed 28 August 2025).

[bibr3-13634593251399224] Bourdieu P (1977) Outline of a Theory of Practice. Cambridge University Press.

[bibr4-13634593251399224] Bourdieu P (1984) Distinction. Routledge and Kegan Paul.

[bibr5-13634593251399224] Bourdieu P (1986) The forms of capital. In: Richardson J (ed.) Handbook of Theory and Research for the Sociology of Education. Greenwood, pp.251–258.

[bibr6-13634593251399224] Bourdieu P (1992) The Logic of Practice. Polity.

[bibr7-13634593251399224] Bourdieu P (1996) The State Nobility. Polity.

[bibr8-13634593251399224] Bourdieu P (1998) Practical Reason: On the Theory of Action. Polity.

[bibr9-13634593251399224] Bourdieu P Passeron C (1996) Reproduction in Education, Society and Culture. Sage.

[bibr10-13634593251399224] Bourdieu P Waquant L (1992) An Invitation to Reflexive Sociology. University of Chicago Press.

[bibr11-13634593251399224] Braun V Clarke V (2006) Using thematic analysis in psychology. Qualitative Research in Psychology 3(2): 77–101.

[bibr12-13634593251399224] British Medical Association (2023) Mental health pressures in England. Available at: https://www.bma.org.uk/advice-and-support/nhs-delivery-and-workforce/pressures/mental-health-pressures-data-analysis (accessed 29 February 2024).

[bibr13-13634593251399224] Callaghan JE Fellin LC Warner-Gale F (2017) A critical analysis of child and adolescent mental health services policy in England. Clinical Child Psychology and Psychiatry 22(1): 109–127. 27052891 10.1177/1359104516640318

[bibr14-13634593251399224] Children and Young People’s Mental Health Coalition (2023) Children and young people’s mental health: An independent review into policy success and challenges over the last decade Available at: https://cypmhc.org.uk/publications/children-and-young-peoples-mental-health-an-independent-review-in-policy-success-and-challenges-over-the-last-decade/ (accessed 29 February 2024).

[bibr15-13634593251399224] Creswell JW Clark VLP (2017) Designing and Conducting Mixed Methods Research. Sage.

[bibr16-13634593251399224] Crossley N (2003) From reproduction to transformation: Social movement fields and the radical habitus. Theory Culture & Society 20(6): 43–68.

[bibr17-13634593251399224] Deer C (2008) Doxa. In: Grenfell M (ed.) Pierre Bourdieu: Key concepts. Acumen, pp.119–130.

[bibr18-13634593251399224] Department of Health (2015) Future in mind: Promoting, protecting and improving our children and young people’s mental health and wellbeing. Available at: https://assets.publishing.service.gov.uk/media/5a80b26bed915d74e33fbe3c/Childrens_Mental_Health.pdf (accessed 29 February 2024).

[bibr19-13634593251399224] Department of Health and Department for Education (2018) Government response to the consultation on transforming children and young people’s mental health provision: A green paper and next steps. Available at: https://www.gov.uk/government/consultations/transforming-children-and-young-peoples-mental-health-provision-a-green-paper (accessed 29 February 2024).

[bibr20-13634593251399224] Dopson S (2009) Changing forms of managerialism in the NHS: Hierarchies, markets and networks. In: Gabe J Calnan M (eds) The New Sociology of the Health Service. Routledge, pp.37–55.

[bibr21-13634593251399224] Durose C Richardson L (2015) Designing Public Policy for Co-production: Theory, Practice and Change. Policy Press.

[bibr22-13634593251399224] Fairclough N (2003) Analysing Discourse: Textual Analysis for Ssocial esearch. Routledge.

[bibr23-13634593251399224] Ferlie E Geraghty KJ (2005) Professionals in public service organizations. In: Ferlie E Ynn L Pollitt LC (eds) The Oxford Handbook of Public Management. Oxford University Press, pp.442–445.

[bibr24-13634593251399224] Fletcher AJ (2017) Applying critical realism in qualitative research: Methodology meets method. International Journal of Social Research Methodology 20(2): 181–194.

[bibr25-13634593251399224] Friedman S Laurison D (2019) The Class Ceiling: Why It Pays to be Privileged. Bristol University Press.

[bibr26-13634593251399224] HM Government (2017) The Government’s response to the five year forward view for mental health. Available at: https://assets.publishing.service.gov.uk/government/uploads/system/uploads/attachment_data/file/582120/FYFV_mental_health__government_response.pdf (accessed 29 February 2024).

[bibr27-13634593251399224] Hudson B Hunter D Peckham S (2019) Policy failure and the policy-implementation gap: Can policy support programs help? Policy Design and Practice 2(1): 1–14.

[bibr28-13634593251399224] Loyal S Quilley S (2017) The particularity of the universal: Critical reflections on Bourdieu’s theory of symbolic power and the state. Theory and Society 46(5): 429–462.

[bibr29-13634593251399224] McDaid D Park AL (2022) The economic case for investing in the prevention of mental health conditions in the UK (Summary). London School of Economics and Political Science/Mental Health Foundation. Available at: https://www.mentalhealth.org.uk/sites/default/files/2022-06/MHF-Investing-in-Prevention-Report-Summary.pdf. (accessed 22 April 2024).

[bibr30-13634593251399224] Mental Health Taskforce (2016) The five year forward view for mental health. Available at: https://www.england.nhs.uk/wp-content/uploads/2016/02/Mental-Health-Taskforce-FYFV-final.pdf (accessed 29 February 2024).

[bibr31-13634593251399224] Metz A Jensen T Farley A , et al (2022) Is implementation research out of step with implementation practice? Pathways to effective implementation support over the last decade. Implementation research and practice 3: 26334895221105585. 10.1177/26334895221105585 PMC9978647 37091077

[bibr32-13634593251399224] National Audit Office (2018a) Financial sustainability of local authorities 2018. Hc: 834, Session 2017–2019. Ministry of Housing, Communities and Local Government London. Available at: https://www.nao.org.uk/reports/financial-sustainability-of-local-authorities-2018/?nab=0 (accessed 26 March 2025).

[bibr33-13634593251399224] National Audit Office (2018b) Improving children and young people’s mental health services. London: National Audit Office. Available at: https://www.nao.org.uk/wp-content/uploads/2018/10/Improving-children-andyoung-peoples-mental-health-services.pdf (accessed 29 February 2024).

[bibr34-13634593251399224] NHS England (2021) Mental health support in schools and colleges. Available at: https://www.england.nhs.uk/mental-health/cyp/trailblazers/ (accessed 29 February 2024).

[bibr35-13634593251399224] NHS England (2023) Mental health of children and young people in England, 2023 - Wave 4 follow up to the 2017 survey. Available at: https://digital.nhs.uk/data-and-information/publications/statistical/mental-health-of-children-and-young-people-in-england/2023-wave-4-follow-up (accessed 29 February 2024).

[bibr36-13634593251399224] Osborne SP (2010) The New Public Governance: Emerging Perspectives on the Theory and Practice of Public Governance. Routledge.

[bibr37-13634593251399224] Passey A (2020) The impacts of co-production on public professionalism: A Bourdieu-inspired analysis of mental health services for children and young people. PhD Thesis, Leeds Beckett University, UK. 10.25448/lbu.25062323.v1

[bibr38-13634593251399224] Pilgrim D (2012) The British welfare state and mental health problems: The continuing relevance of the work of Claus Offe. Sociology of Health & Illness 34(7): 1070–1084. 22530616 10.1111/j.1467-9566.2011.01447.x

[bibr39-13634593251399224] Reay D (2017) Miseducation: Inequality, Education and the Working Classes. Bristol University Press.

[bibr40-13634593251399224] Rowlands J Rawolle S (2013) Neoliberalism is not a theory of everything: A Bourdieuian analysis ofillusioin educational research. Critical Studies in Education 54(3): 260–272.

[bibr41-13634593251399224] Royal College of Psychiatrists (2024) Available at: https://www.rcpsych.ac.uk/news-and-features/latest-news/detail/2024/02/07/we-cannot-allow-childhood-mental-illness-to-become-the-new-norm—rcpsych (accessed 29 February 2024).

[bibr42-13634593251399224] Savage M (2015) Social Class in the 21st Century. Penguin.

[bibr43-13634593251399224] Standing G (2011) The Precariat. Bloomsbury.

[bibr44-13634593251399224] Storey J Holti R Hartley J , et al (2019) Devolving healthcare services redesign to local clinical leaders: Does it work in practice? Journal of Health Organization and Management 33(2): 188–203. 30950312 10.1108/JHOM-05-2018-0144

[bibr45-13634593251399224] Thomson P (2005) Bringing Bourdieu to policy sociology: Codification, misrecognition and exchange value in the UK context. Journal of Education Policy 20(6): 741–758.

[bibr46-13634593251399224] Waller R Ingram N Ward RM (2017) Higher Education and Social Inequalities: University Admissions, Experiences and Outcomes. Routledge.

[bibr47-13634593251399224] World Health Organization (2021) Mental health of adolescents. Available at: https://www.who.int/news-room/fact-sheets/detail/adolescent-mental-health (accessed 29 February 2024).

